# A phoenix in the greenhouse: characterization and phylogenomics of complete chloroplast genomes sheds light on the putatively extinct-in-the-wild *Solanum ensifolium* (Solanaceae)

**DOI:** 10.1186/s12870-025-06338-8

**Published:** 2025-03-12

**Authors:** Matthew R. Graham, Noorpreet Kaur, Cynthia S. Jones, Kurt Lamour, Bryan A. Connolly

**Affiliations:** 1 Department of Biology, Eastern Connecticut State University, Willimantic, CT 06226 USA; 2 Department of Ecology and Evolutionary Biology, University of Connecticut, Storrs, CT 06269 USA; 3 Department of Entomology and Plant Pathology, University of Tennessee, Knoxville, TN 37996 USA

**Keywords:** Caribbean flora, Endangered species, Evolutionary biology, Plant genomics, Systematics, Solanaceae

## Abstract

**Background:**

The genus *Solanum* is a diverse group of flowering plants with significant economic importance. Within this genus, the subgenus *Leptostemonum*, comprising spiny solanums, is particularly noteworthy due to its high species diversity and endemism. *Solanum ensifolium*, a member of this subgenus, is a critically endangered species endemic to Puerto Rico and known locally as erubia. The species survives in greenhouses and botanical gardens and is thought to be extinct in the wild, but with reintroduction efforts in progress. Despite its conservation status, genomic data for *S. ensifolium* remains scarce, limiting our understanding of its evolutionary history and potential adaptations.

**Results:**

The *S. ensifolium* chloroplast genome (155,295 bp) exhibits a typical quadripartite structure and encodes 151 genes, including 95 protein-coding genes involved in photosynthesis, transcription, translation, and other essential cellular functions. Gene content and genome organization are similar to those observed in closely related *Solanum* species. Comparative genomic analysis of the annotated genome with that of closely related *Solanum* species revealed differences in nucleotide diversity between the large single-copy (LSC) and small single-copy regions (SSC), and the inverted repeat (IR) regions. Additionally, phylogenetic analyses confirmed placement of *S. ensifolium* within the *Leptostemonum* subgenus, affirming its suspected close relationship with *S. crotonoides* and *S. aturense*. Furthermore, of the three individuals of *S. ensifolium* for which chloroplast genomes were obtained, no genetic variation was observed.

**Conclusions:**

The availability of the *S. ensifolium* chloroplast genome provides insights into its evolutionary history and conservation needs. Comparative genomics uncovered evolutionary differences in *Solanum* chloroplast genomes, including nucleotide diversity and structural variations. Phylogenetic analyses confirmed the close relationship between *S. ensifolium* and other *Leptostemonum* species. These findings enhance our understanding of this critically endangered species' evolution, guiding effective conservation strategies like using chloroplast variation to assess genetic diversity for ex situ conservation and reintroduction programs. The uniformity of the chloroplast genome in *S. ensifolium* may reveal that this species has undergone a genetic bottleneck. To prevent inbreeding depression and maintain evolutionary adaptability, efforts should be made to generate and preserve as much genetic diversity as possible.

**Supplementary Information:**

The online version contains supplementary material available at 10.1186/s12870-025-06338-8.

## Background

Understanding the evolutionary history of plant lineages with complex diversification histories is crucial for conservation efforts. Chloroplast genomes, encoding essential genes for photosynthesis and other cellular functions, are valuable tools in plant systematics and evolution due to their unique structure, history, and ease of sequencing [9]. Advancements in sequencing technologies have further empowered this approach by enabling the generation of large-scale chloroplast genome datasets, which have led to significant progress in understanding plant diversity, evolution, and conservation [8, 16, 18, 34]. These advantages make chloroplast genomes particularly well-suited for studying plant species with complex evolutionary histories and diverse lineages, such as the genus *Solanum*.

The *Solanum* genus, belonging to the Solanaceae family, is a prominent group of flowering plants renowned for its economic significance [15]. It encompasses a remarkable range of morphological and ecological variation, with estimates suggesting over 1,500 species. *Solanum* species have profoundly shaped human civilization, providing staple food crops like potato (*S. tuberosum*) and tomato (*S. lycopersicum*), as well as several minor crops such as eggplant (*S. melongena*) and pepino (*S. muricatum*). Subgenus *Leptostemonum*, also called the spiny solanums, is the most diverse major *Solanum* lineage, comprising about a third of the species in the genus. The center of diversity for spiny solanums lies in Central and South America, with a significantly higher number of species compared to their presence in the Old World; Africa, Madagascar, and Australia [30]. New *Solanum* species within this subgenus are still being regularly discovered (i.e. [23]).

**Fig. 1:**
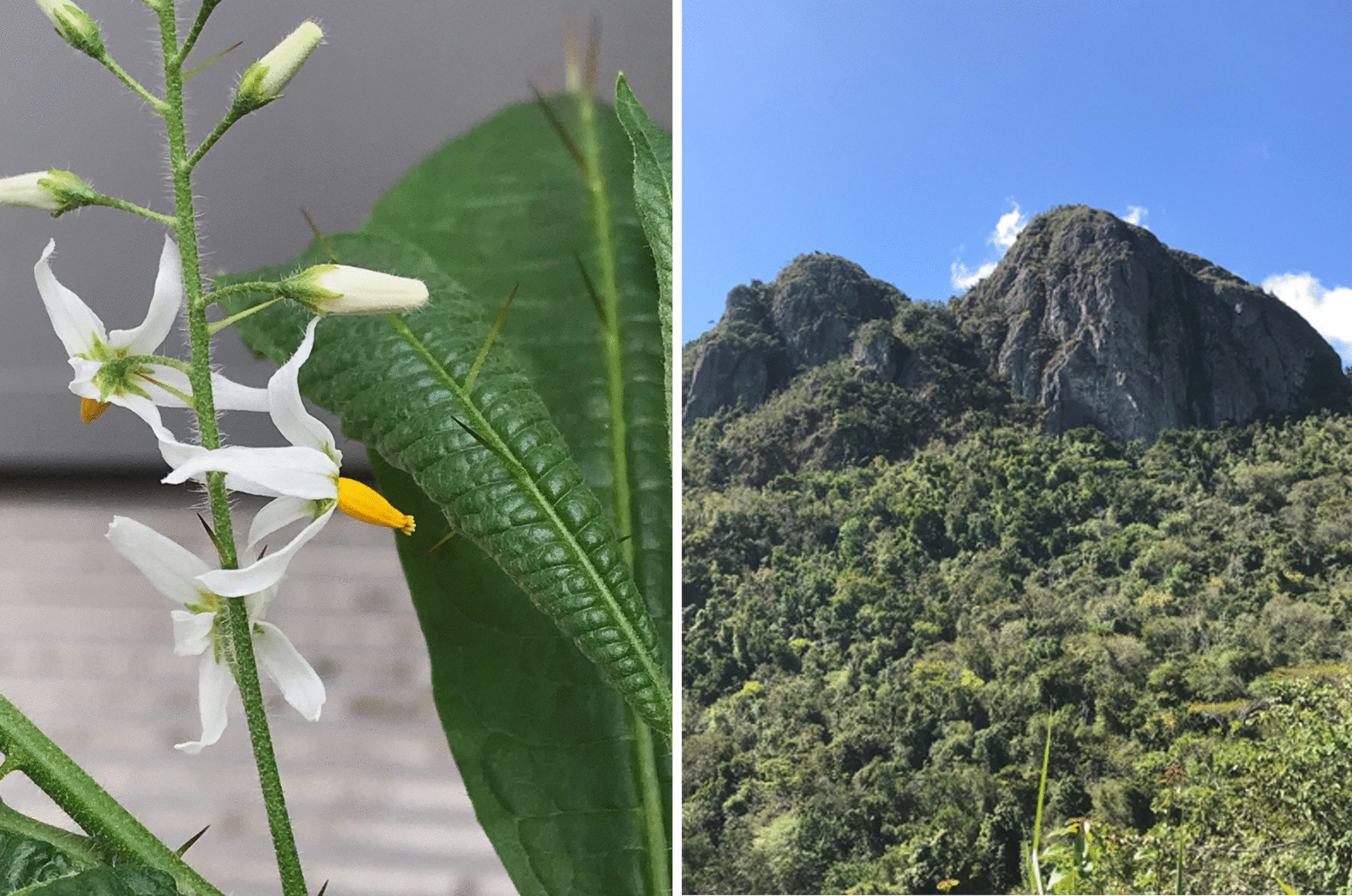
*Solanum ensifolium* (Erubia) flowers and leaves (left) and its former forest habitat in Puerto Rico (right)

Of these, the small *Solanum bahamense* L. species group is endemic to the Carribbean, growing up to 4.5 m tall in shoreline and montane scrub habitats. The group was rendered monophyletic and contains three species: S*. bahamense* L. found throughout the Caribbean archipelago, *S. polyacanthos* Lam. from Hispaniola, and *S. ensifolium* Dunal (= *S. drymophilum* O.E. Schulz) endemic to Puerto Rico [27]. *Solanum bahamense* and *S. polyacanthos* are not listed as threatened, but *S. ensifolium*, known locally as erubia, is critically endangered and thought to be extinct in the wild due to habitat loss [25]. Historically, its distribution was confined to specific forest habitats on the north central and southeast parts of the island (Fig. 1). Most recent records are from the municipalities of Salinas and Arecibo. Despite its precarious status, genomic data for *S. ensifolium* remains scarce. Particularly, complete chloroplast genome sequences are lacking for the *S. bahamense* species group but could offer valuable insights into its evolution and conservation strategies.

For example, Huang et al. [17] demonstrated the power of chloroplast genome analysis for resolving complex phylogenetic relationships within the *Solanum* genus, as exemplified by their study on *Solanum* section *Petota*. Caycho et al. [6] highlighted the importance of characterizing chloroplast genomes for understudied plant species, emphasizing the value of such data for future studies regarding diversity or genetic improvement. Furthermore, Li et al. [22] demonstrated the potential of chloroplast genome data for phylogenetic analysis of orchid genus *Pholidota* and identified mutational hotspots that could be used as molecular markers.

Inspired by these studies, we address this critical knowledge gap for *S. ensifolium* by assembling and analyzing the complete chloroplast genome using tissue samples from greenhouses.

This study follows a similar approach to Caycho et al. [6] and Li et al. [22] by employing de novo assembly, annotation, and comparative analyses of the chloroplast genome. By providing the first complete chloroplast genome sequence for this species, we offer an opportunity to explore the evolution of this essential organelle and gain deeper insights into the evolutionary history of *S. ensifolium* itself. Additionally, the chloroplast genome provides a foundation for estimating genetic diversity among individuals, which could be crucial for conserving the critically endangered *S. ensifolium.*

## Results

### First report of the *Solanum ensifolium* chloroplast genome: assembly and annotation

**Fig. 2:**
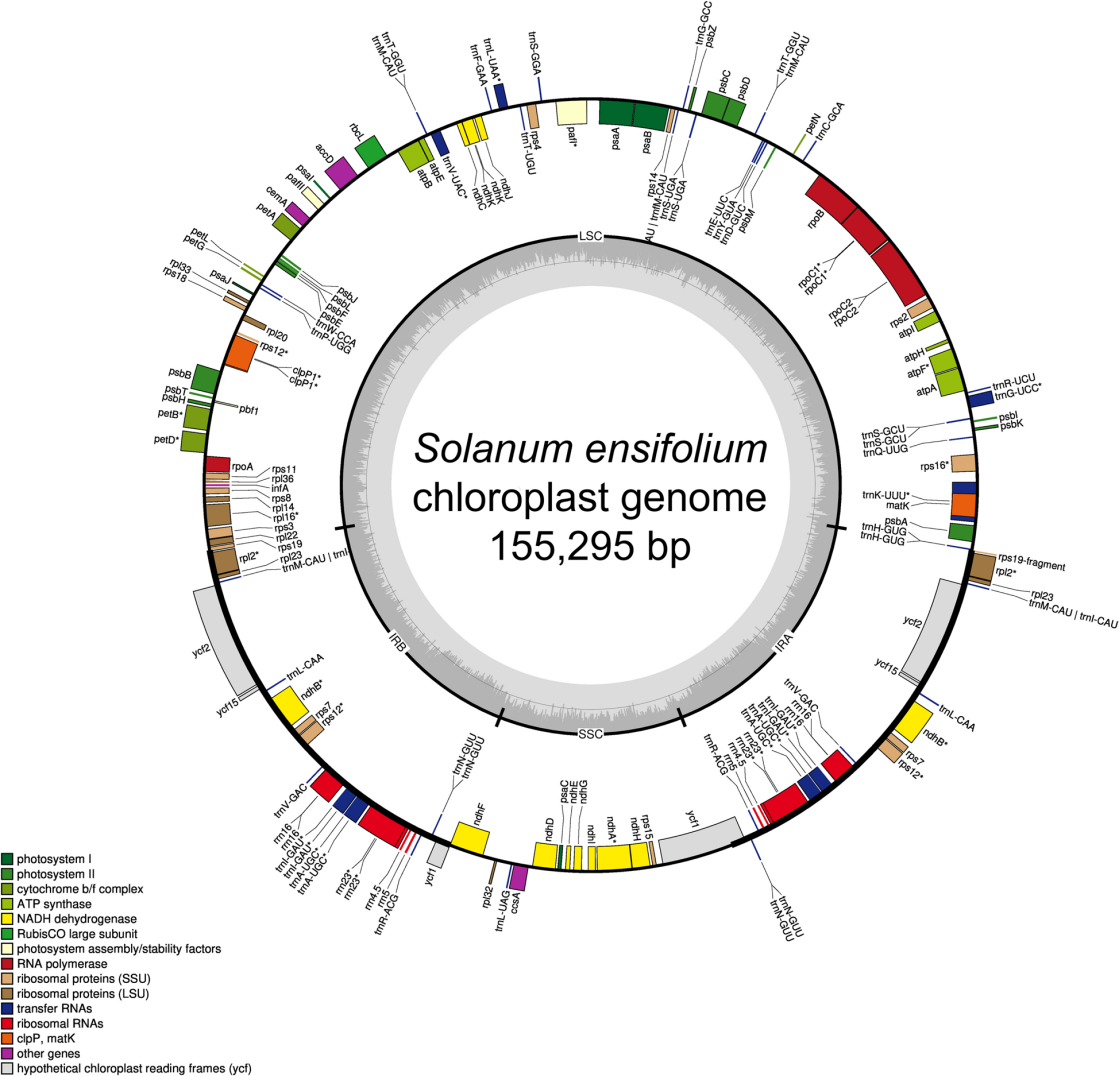
Circular representation of the *Solanum ensifolium* chloroplast genome. The four genomic regions (LSC, SSC, IRA, and IRB) are depicted. Genes inside the circle are transcribed clockwise, while those outside are transcribed counter-clockwise. Functional groups of genes are color-coded. The inner circle displays GC content (dark gray) and AT content (light gray). Similar diagrams are presented for *S. aturense* and *S. crotonoides* in Fig. S1

We assembled the chloroplast genome for three *S. ensifolium* samples. Genomes were identical with a length of 155,295 bp, GC content of 37.5%, and were arranged in the typical quadripartite structure (Fig. 2): long single copy sequence (LSC) of 86,012 bp (~ 55.39% of the genome), a short single copy sequence (SSC) 18,417 bp in length (~ 11.86%), and two inverted repeat regions (IRs) of 25,433 bp in length (~ 16.38%). A total of 151 genes were identified in the *S. ensifolium* genome, including 95 protein-coding genes, 52 tRNA-coding genes, and 12 rRNA-coding genes. These numbers are similar to those found in *Solanum aturense* and *Solanum crotonoides* (Fig. S1), the most phylogenetically closely related species for which a chloroplast genome is available (Tables 1 and 2).

**Table 1:** Comparison of chloroplast genome features of *S. ensifolium* to *S. crotonoides*

Species	Size (bp)	Total GC (%)	LSC (bp)	SSC (bp)	IRs (bp)	No. Genes	PCGs	tRNA Genes	rRNA Genes	No. Genes w/ Introns
*S. ensifolium*	155,295	37.5	86,012	18,417	25,433	151	95	52	12	19
*S. crotonoides*	153,797	37.8	84,583	18,471	25,326	148	94	50	12	18

**Table 2:** *Solanum ensifolium* chloroplast genes classified by functional category and subgroup

Category	Functional Group	Annotated Genes
Transcription & Translation	Large subunit of ribosomal proteins	*rpl*2″*, *rpl*14, *rpl*16*, *rpl*20, *rpl*22, *rpl*23*, *rpl*32, *rpl3*3, *rpl*36
	Small subunit of ribosomal proteins	*rps*2, *rps*3, *rps*4, *rps*7″, *rps*8, *rps*11, *rps*12″*, *rps*14, *rps*15, *rps*16*, *rps*18, *rps*19
	DNA dependent RNA polymerase	*rpo*A, *rpo*B, *rpo*C1*, *rpo*C2
	Translation	
	rRNA	*rrn*4.5″, *rrn*5″, *rrn*16″, *rrn*23”
	tRNA	*trn*A-UGC”*, *trn*C-GCA, *trn*D-GUC, *trn*E-UUC, *trn*F-GAA, *trn*G-GCC, *trn*G-UCC*, *trn*H-GUG, *trn*I-CAU”, *trn*I-GAU*”, *trn*K-UUU*, *trn*L-CAA*, *trn*L-UAA*, *trn*L-UAG, *trn*fM-CAU, *trn*M-CAU, *trn*N-GUU*, *trn*P-UGG, *trn*Q-UUG, *trn*R-ACG*, *trn*R-UCU, *trn*S-GCU, *trn*S-GGA, *trn*S-UGA, *trn*T-GGU, *trn*T-UGU, *trn*V-GAC*, *trn*V-UAC*, *trn*W-CCA, *trn*Y-GUA
Photosynthesis	Photosystem I	*psa*A*, psa*B*, psa*C*, psa*I, *psa*J
	Photosystem II	*psb*A*, psb*B, *psb*C, *psb*D, *psb*E, *psb*F, *psb*H, *psb*I, *psb*J, *psb*K, *psb*L, *psb*M, p*sb*Z
	NADH dehydrogenase	*ndh*A*, ndhB”*, *ndh*C, *ndh*D, *ndh*E, *ndh*F, *ndhG*, *ndh*H, *ndh*I, *ndh*J, *ndh*K
	Cytochrome b6/f complex	*pet*A, *pet*B*, *pet*D*, *pet*G, *pet*L, *pet*N
	ATP synthase	*atp*A, *atp*B, *atp*E, *atp*F*, *atp*H, *atp*I
	Rubisco	*rbc*L
Other genes	Maturase	*mat*K
	Protease	*clp*P*^
	Envelope membrane protein	*cem*A
	Subunit Acetyl-CoA-Carboxylase	*acc*D
	c-type cytochrome synthesis gene	*ccs*A
	Prolamin-box binding factor 1 gen	*pbf*1
Unknown	Putative essential protein	*ycf*1″, *ycf*2″, *ycf*3 (*paf*I)”*, *ycf*4 (*paf*II)

”Duplicated genes

*Genes with introns

^Possible pseudogenes

The annotated *S. ensifolium* chloroplast genome encompassed 12 small ribosomal proteins (*rps*) and 9 large ribosomal proteins (*rpl*), essential for protein synthesis. It included components for transcription and translation, such as 4 DNA-dependent RNA polymerases (*rpo*), 4 rRNA (*rrn*), 30 tRNA (*trn*), and translation initiation factor 1 (*inf*A). The genome also encoded proteins essential for photosynthesis, including 5 photosystem I proteins (*psa*), 13 photosystem II proteins (*psb*), 11 NADH dehydrogenase proteins (*ndh*), 6 cytochrome b6/f complex proteins (*pet*), and 6 ATP synthase complex proteins (*atp*). Additionally, metabolic enzymes like Rubisco (*rbc*L), beta subunit of acetyl-CoA carboxylase (*acc*D), and cytochrome C biogenesis protein (*ccs*A) were present. Protein processing and degradation functions were represented by maturase K (*mat*K), proteolytic subunit of ATP-dependent Clp protease (*clp*P), and membrane envelope protein (*cem*A). The genome contained 4 hypothetical proteins of unknown function (*ycf*).

### Codon usage analysis

**Fig. 3:**
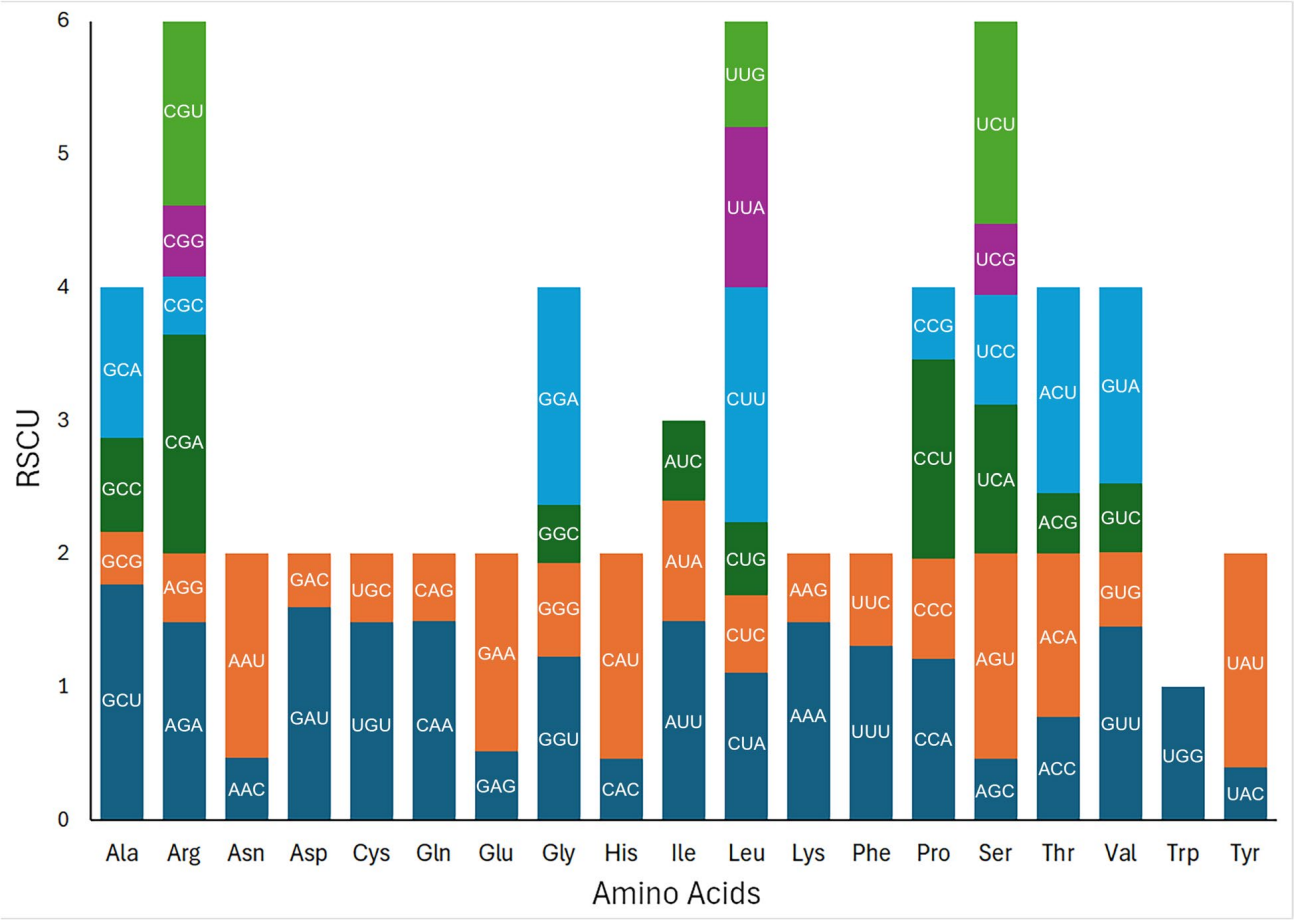
Relative Synonymous Codon Usage (RSCU) in *Solanum ensifolium* chloroplast genes

Codon frequency and Relative Synonymous Codon Usage (RSCU) were calculated for all protein-coding regions (Fig. 3). A total of 64 codons were found in the chloroplast genome of *S. ensifolium*. AUU (Isoleucine, *n* = 1,239) was the most prevalent, followed by AAA (Lysine, *n* = 1,185), GAA (Glutamic acid, *n* = 1,148), AAU (Isoleucine, *n* = 1,126), and UUU (Phenylalanine, *n* = 1,069). Stop codons UGA (*n* = 25), UAG (*n* = 26), and UAA (*n* = 49) were the least frequent. Among amino acid-encoding codons, UGC (Cysteine, *n* = 91), CGC (Arginine, *n* = 120), and AGC (Serine, *n* = 137) exhibited the lowest occurrences.

Of the 64 codons, 30 had RSCU values > 1, all of which ended in A or U. The RSCU values for 32 codons were < 1, 30 of which ended with base C or G. Methionine (AUG) and Tryptophan (UGG) were uniquely encoded, indicating no codon usage bias for these amino acids (RSCU = 1).

### Identification of repetitive sequences

**Fig. 4:**
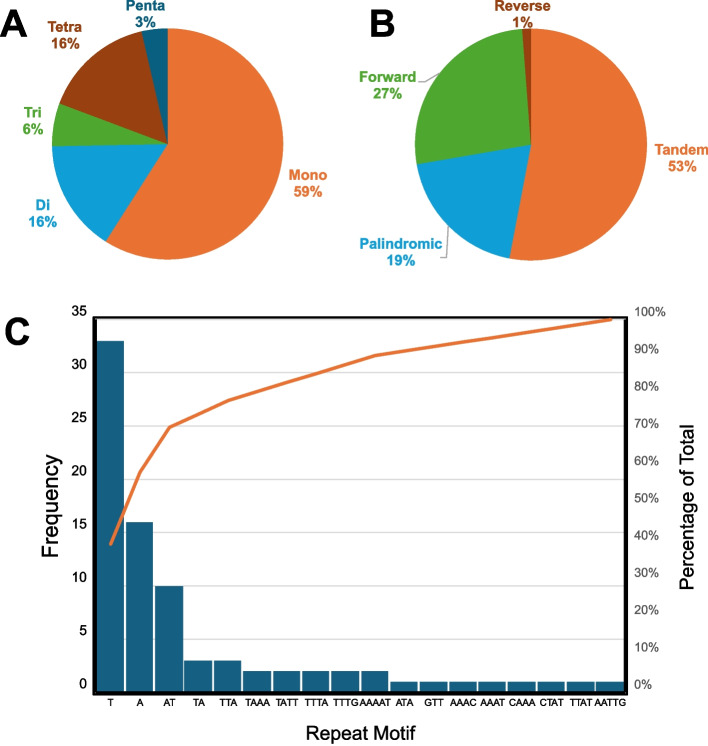
Summary of the distribution of SSRs identified in the *Solanum ensifolium* plastome. **A** Percentage of SSRs with different motif lengths (mono-, di-, tri-, tetra-, and penta-nucleotides). **B** Distribution of SSRs according to their orientation (tandem, palindromic, forward, and reverse). **C** Frequency of each repeat motif, with an orange line indicating the cumulative percentage of SSRs

Simple Sequence Repeat (SSR) analysis uncovered 83 loci, encompassing 49 single-base, 13 dinucleotide, 5 trinucleotide, 14 tetranucleotide, and 3 pentanucleotide motifs (Fig. 4A). Repeat motif patterns revealed a predominance of tandem repeats (44, ~ 53%), followed by palindromic (16, ~ 19%), forward (22, ~ 27%), and a single reverse repeat (~ 1%) (Fig. 4B). The most prevalent repeat motifs were T, A, and AT (Fig. 4C).

### Phylogenetic relationships among *Solanum* species

**Fig. 5:**
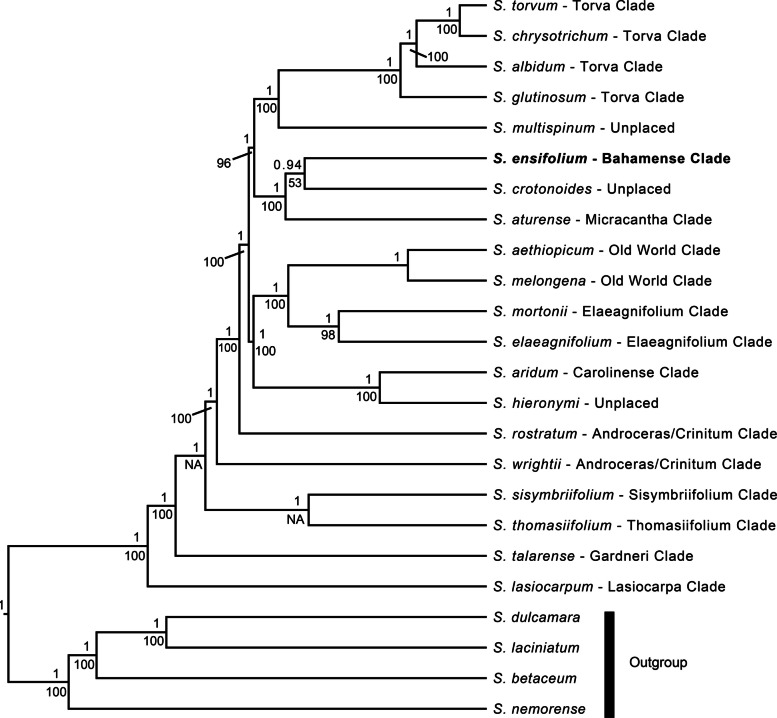
Ultrametric phylogeny of *Solanum* chloroplast genomes, inferred using Bayesian inference (BEAST) with posterior probabilities (PP) above branches and bootstrap percentages (BS) below branches. *Solanum ensifolium* (in bold), for which the complete chloroplast genome was sequenced in this study, is classified within the Bahamense Clade. Ingroup species (Subgenus *Leptostemonum*) are designated by their clade affiliation following the species name. "NA" indicates branches not recovered in the maximum likelihood analysis (see Fig. S2)

Phylogenetic analyses employing Maximum Likelihood (ML) and Bayesian Inference (BI) yielded largely congruent *Solanum* chloroplast genome trees (Figs. 5 & S2). Most nodes exhibited robust support under both analytical frameworks. However, the sister relationship between *S. ensifolium* and *S. crotonoides*, while strongly supported by BI (posterior probability = 0.94), received weaker support from ML (bootstrap support = 53). These two species formed a monophyletic group with *S. aturense*, a clade strongly supported by both analyses. The Torva, Old World, and *Elaeagnifolium* clades were consistently well-supported, whereas the Androceras/Crinitum clade appeared paraphyletic.

### Genomic comparison to *Solanum crotonoides*

Given that our Bayesian phylogenetic analysis grouped *S. crotonoides* as the most closely related species to *S. ensifolium* (for which chloroplast genome data are available), we conducted comparative analyses of these two species.

**Fig. 6:**
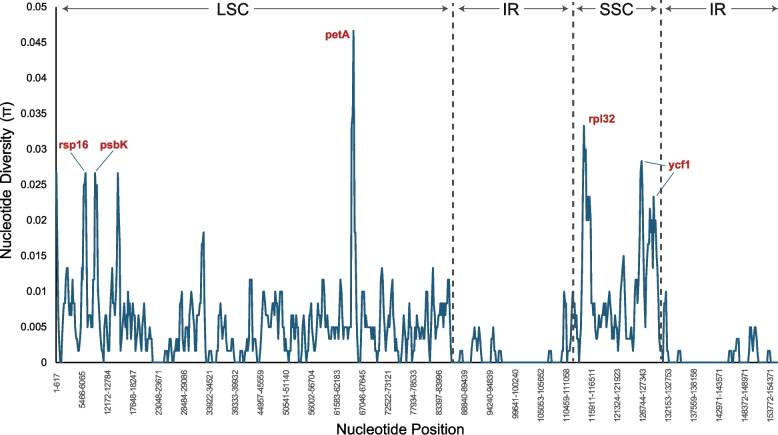
Sliding window analysis of nucleotide diversity (π) in *Solanum ensifolium* and *S. crotonoides* complete plastomes. The window size is 600 bp, and the step size is 200 bp

Sliding window analysis revealed varying levels of nucleotide diversity across the chloroplast genome (Fig. 6). The LSC region exhibited an average Pi of 0.006, while the SSC region displayed a higher average Pi of 0.011. In contrast, the IR regions demonstrated significantly lower nucleotide diversity with an average Pi of 0.001. These results indicate that the IR regions are more conserved than the single-copy regions (LSC and SSC), consistent with the generally accepted pattern of chloroplast genome evolution. Notably, regions including rsp16, psbK, petA, rpl32, and ycf1 exhibited the highest nucleotide diversity.

**Fig. 7:**
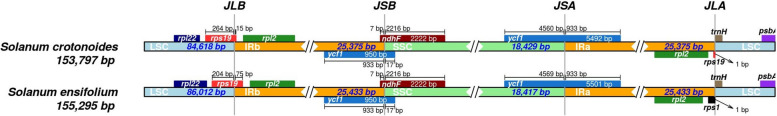
Visual comparisons of the borders between the large single-copy (LSC), small single-copy (SSC), and inverted repeat (IR) regions for the complete chloroplast genomes of *Solanum ensifolium* and *S. crotonoides*. The junctions where LSC and SSC meet the inverted repeat regions (IRa and IRb) are labeled: JLA for LSC/IRa, JLB for LSC/IRb, JSA for SSC/IRa, and JSB for SSC/IRb. Colored boxes depict genes, with numbers above or below indicating the distance in base pairs between a gene's end and the closest border

Boundary shift analysis of chloroplast genome regions (Fig. 7) revealed identical positions of the IRb/SSC (JSB) and SSC/IRa (JSA) boundaries between *S. ensifolium* and *S. crotonoides*. However, a 60 bp displacement was observed at the LSC/IRb junction (JLB) in *S. ensifolium* compared to *S. crotonoides*. Additionally, a single base pair difference was detected at the IRa/LSC boundary (JLA) between the two species.

## Discussion

### Comparative genomics and evolutionary relationships

The comparative genomic analysis between *S. ensifolium* and *S. crotonoides* provides some insight into the evolution of chloroplast genomes within the *Solanum* genus. The observed differences in nucleotide diversity between the single-copy regions (LSC and SSC) and the inverted repeat (IR) regions highlight possible evolutionary pressures acting on these genomic compartments. Specifically, the higher nucleotide diversity observed in the single-copy regions suggests that these regions may be more susceptible to genetic drift and selection, potentially leading to the accumulation of adaptive mutations. This increased variability could be attributed to relaxed selective constraints, adaptive evolution, or recombination in these regions. For example, genes within the single-copy regions might be less constrained by purifying selection due to functional redundancy or the presence of regulatory elements that buffer the effects of mutations. Alternatively, some genes might be involved in adaptive processes, leading to higher rates of nucleotide substitution. Recombination, although rare in chloroplasts [28], could also contribute to increased genetic diversity in the single-copy regions.

To further explore the adaptive significance of the observed nucleotide diversity, future studies could focus on identifying specific genes or regions within the LSC and SSC that exhibit elevated levels of variation. Functional analyses of these genes could provide insights into their potential roles in adaptation or other biological processes. For example, gene expression studies could reveal whether genes with high nucleotide diversity are differentially expressed under specific environmental conditions or developmental stages.

Additionally, the comparative analysis revealed structural variations between the chloroplast genomes of *S. ensifolium* and *S. crotonoides*, particularly in the LSC/IRb junction. These variations might have implications for gene expression or genome organization. For example, changes in the LSC/IRb junction (Fig. 7) could affect the expression of genes located near this boundary, potentially disrupting gene expression patterns.

To elucidate the functional consequences of these structural variations, further investigations are needed. Comparative transcriptomic analysis, for instance, could reveal differences in gene expression patterns between *S. ensifolium* and *S. crotonoides* that are associated with the structural variations. Additionally, computational modeling and functional assays could be used to investigate the potential impact of these variations on chloroplast genome stability and function.

### Implications for conservation

The availability of the *S. ensifolium* chloroplast genome sequence offers potentially valuable insights for conservation efforts, particularly considering its critically endangered status and potential extinction in the wild. By comparing the chloroplast genome to closely related species and monitoring genetic diversity within greenhouse populations over time, researchers could identify unique genetic markers and assess genetic erosion using chloroplast variation as a proxy for overall genetic diversity [11]. Although the initial analysis of three *S. ensifolium* individuals revealed identical chloroplast genomes, additional sampling of additional ex situ specimens, or undiscovered wild populations, could uncover intraspecific genetic diversity. This would be beneficial for developing effective conservation strategies, as genetic diversity is essential for the long-term survival of species.

By analyzing chloroplast variation to estimate genetic diversity, conservation biologists could develop targeted conservation strategies for *S. ensifolium*, such as ex situ germplasm banking and reintroduction programs. Ex situ germplasm banking involves the collection and storage of genetic material of plant species outside their natural habitat. For example, by exploiting their natural desiccation tolerance, seeds of imperiled plant species can be effectively stored in freezers or cryogenic platforms [31]. For *S. ensifolium*, this would involve preserving genetic material, especially seeds, from greenhouse-grown individuals. By maintaining a diverse collection of genetic material, ex situ germplasm banks can safeguard the genetic diversity of the species and provide a source of material for reintroduction efforts. Currently, seeds of *S. ensifolium* have been banked at the Atlanta Botanic Garden and germplasm was used for re-introduction efforts during the spring of 2024 conducted by Para La Naturaleza and U.S. Fish and Wildlife Service in collaboration with author BAC.

The availability of the *S. ensifolium* chloroplast genome sequence can also facilitate the development of efficient methods for germplasm storage and management. Genetic markers derived from the chloroplast genome can be used to track the genetic diversity of stored samples and to identify individuals with unique genetic characteristics. This information could be used to optimize germplasm storage conditions and to select individuals for reintroduction programs.

## Conclusion

Comparative genomic analysis between *S. ensifolium* and *S. crotonoides* provided insights into the evolution of chloroplast genomes within the *Leptostemonum* subgenus. The observed differences in nucleotide diversity and structural variation suggests that distinct evolutionary pressures must be acting on different genomic regions. These findings have implications for conservation efforts, particularly for the critically endangered *S. ensifolium*. Furthermore, the availability of the *S. ensifolium* chloroplast genome sequence enables the development of targeted conservation strategies, such as ex situ germplasm banking and reintroduction programs. By leveraging genetic information from the chloroplast genome, researchers can effectively assess and monitor genetic diversity, optimize germplasm storage, and select individuals for reintroduction efforts, thereby contributing to the long-term survival of the species. If the initial introductions are as successful as would be hoped, then long-term survival in natural populations will also depend on understanding natural reproduction, via vegetative propagation, and natural pollination (e.g., [2]).

## Methods

### Plant materials, DNA extraction, and sequencing

Three genotypes of *Solanum ensifolium* that originated from Las Piedras del Collado in the municipality of Salina, Puerto Rico were maintained ex situ at Fairchild Tropical Botanical Garden and greenhouses at University of Connecticut, University of Utah, and Eastern Connecticut State University for over 25 years. For tracking purposes, these genotypes were labelled A, B, and U. Fresh leaves of each genotype were collected and placed in small food stage bags and shipped on ice to author KL for DNA extraction and sequencing.

Leaves of three individual *S. ensifolium* were removed from greenhouse specimens and used for DNA extraction using a DNEasy Plant Extraction Kit (Qiagen, Hilden, Germany). Leaf discs were lyophilized in 2 ml screw top microfuge tubes preloaded with four 3 mm glass beads, bashed to a fine powder on a mixer-mill device (Qiagen, Hilden, Germany) followed by the addition of the initial AP1 lysis buffer and all steps following were according to the manufacturer’s instructions.

The Illumina libraries were constructed using total DNA from each sample and a PCR-free workflow using a KAPA Hyperprep kit according to manufacturer directions [32]. Libraries were then sent to Admera Health LLC (Plainfield, New Jersey, USA) for sequencing on a NovaSEQ device running a 2 × 150 paired-end configuration. FastQC (Babraham Institute, https://www.bioinformatics.babraham.ac.uk/projects/fastqc/) was employed as an initial step in the pre-assembly process to assess read quality. It provided a summarized overview of key quality metrics, including per-base PHRED scores, the average occurrence of ‘N’ bases (i.e., undefined bases), GC content, read length distributions, identification of overrepresented sequences, and the detection of adapter sequences. Next, Trimmomatic [5] was used to eliminate adapter sequences and low-quality bases and reads from paired-end libraries.

### Chloroplast genome assembly, annotation, and characterization

The *S. ensifolium* chloroplast genome was assembled using the Illumina reads through two complementary approaches. First, a reference-based assembly was performed in Geneious Prime by mapping the reads to the well-annotated *S. melongena* chloroplast genome (GenBank: NC_030207). This approach leverages the existing reference to guide the assembly process and ensure high accuracy in capturing conserved regions. Second, a de novo assembly strategy was employed using NOVOPlasty v.4.2.1 [10]. Here, the *S. melongena* chloroplast genome again served as a reference, but we assigned a single, randomly chosen Illumina read as a seed to initiate the assembly process in NOVOPlasty. This seed-based approach allows for potentially more flexibility in capturing variation present in the *S. ensifolium* chloroplast DNA compared to the reference.

The *S. ensifolium* chloroplast genome was annotated using a combination of computational and manual methods. The GeSeq v2.03 program [29] within the Chlorobox webserver (https://www.mpimp-golm.mpg.de/2168/en) predicted gene locations, referencing the *S. melongena* chloroplast genome (GenBank: NC_030207). This process identified all major features including chloroplast inverted repeats (IRs), rps12 interspersed genes, protein-coding sequences, transfer RNAs (tRNAs), and ribosomal RNAs (rRNAs). A minimum identity threshold of 25% was applied for protein-coding genes and 85% for RNAs to ensure accuracy. Additionally, tRNAscan-SE v2.0.7 [7] on the same server served as a secondary tRNA annotator. Further confirmation was achieved using Chloë v0.1.0, another external Chlorobox annotator. Finally, all annotations were meticulously reviewed and corrected using Geneious Prime v2023.0.4 (Biomatters Ltd., New Zealand). This comprehensive approach ensured high-quality annotation of the *S. ensifolium* chloroplast genome.

Following annotation, A circular chloroplast genome map was constructed using OGDRAW v.1.3.1 [14] based on the annotated genome sequence. The complete *S. ensifolium* chloroplast genome sequence has been deposited in the NCBI GenBank database under accession number PP744563.

### Codon usage

Codon usage analysis was conducted to investigate codon bias and potential translational efficiency in the *S. ensifolium* plastome. The analysis focused on all protein-coding sequences. Relative Synonymous Codon Usage (RSCU) values and codon frequencies were calculated using DAMBE5 v7.3.32 [33]. Higher RSCU values (> 1) indicate codons preferred by the *S. ensifolium* chloroplast, while lower values (< 1) suggest less frequent usage.

### SSR analysis

Simple Sequence Repeat (SSR) analysis was conducted on the *S. ensifolium* plastome to identify dispersed, palindromic, and tandem repeats. REPuter v.2.74 [21] was employed to detect dispersed and palindromic repeats, using a minimum repeat size of 30 bp and a permissive Hamming distance of 3 (at least 90% sequence identity). Tandem repeats were identified using Tandem Repeats Finder v.4.09 [4] with default parameters. Finally, SSR motif identification and classification were performed with MISA v.1.01 [3], applying established minimum repeat thresholds of 10 for mononucleotides, 5 for dinucleotides, and 4 for tri-, tetra-, penta-, and hexanucleotides.

### Phylogenomic analysis

To elucidate the phylogenetic placement of *S. ensifolium* within subgenus *Leptostemonum*, a phylogenomic analysis was conducted using Maximum Likelihood (ML) and Bayesian Inference (BI) approaches. Complete chloroplast genome sequences for the following 19 species of subgenus *Leptostemonum* were downloaded from GenBank (National Center for Biotechnology Information, NCBI): *S. torvum* (NC_085711.1), *S. chrysotrichum* (MZ221889.1), *S. albidum* (MZ221905.1), *S. glutinosum* (MZ221882.1), *S. multispinum* (MZ221923.1), *S. crotonoides* (MZ221878.1), *S. aturense* (NC_062420.1), *S. aethiopicum* (MN218076.1), *S. melongena* (MF818319.1), *S. mortonii* (MZ221922.1), *S. elaeagnifolium* (KX792501.2), *S. aridum* (MZ221914.1), *S. hieronymi* (MZ221883.1), *S. rostratum* (MN635796.1), *S. wrightii* (MN218084.1), *S. sisymbriifolium* (NC_061213.1), *S. thomasiifolium* (MZ221843.1), *S. talarense* (MZ221863.1), and *S. lasiocarpum* (NC_086861.1). Additionally, the following four plastomes from *Solanum* species outside *Leptostemonum* were downloaded as outgroup taxa: *S. dulcamara* (KY863443.1), *S. laciniatum* (MZ221919.1), *S. betaceum* (MN599115.1), and *S. nemorense* (MZ221924.1). These sequences, along with the newly sequenced *S. ensifolium* plastome, were aligned using MAFFT [19] implemented in Geneious Prime.

For the Maximum Likelihood analysis, the aligned plastome sequences were used as input for IQTREE v2.0.6 [24]. The "-m TEST" option was employed to allow the software to determine the best-fitting substitution model, which was determined to be K3Pu + I + G10. This model accounts for different rates of nucleotide substitutions, invariant sites, and rate heterogeneity among sites.The analysis was run using the K3Pu + I + G10 model with 1,000 bootstrap replicates to assess branch support.

For the BI analysis, the aligned sequences were imported into MEGAX [20] to identify a substitution model compatible with BEAST v1.8.0 [13]. Based on this analysis, a GTR + G substitution model was selected for the BEAST analysis. An XML file specifying the data, substitution model, and clock settings (uncalibrated, rate = 1.0) was created using BEAUTi (part of the BEAST package). The BEAST analysis was run for 10 million generations, sampling every 10,000 iterations. Tracer v1.6 [12] was used to confirm that all model parameters achieved effective sample size (ESS) values exceeding 200. TreeAnnotator (part of the BEAST package) was used to generate a maximum credibility consensus tree after discarding the first 2,500 trees as burn-in. The resulting consensus trees from both IQTREE and BEAST were visualized and explored using FigTree v1.4.4 (http://tree.bio.ed.ac.uk/software/). Final tree illustrations were prepared using Adobe Illustrator.

### Comparative chloroplast genomics of *Solanum ensifolium* and *S. crotonoides*

Given the close phylogenetic relationship between *S. ensifolium* and *S. crotonoides* inferred from Bayesian analysis, we conducted comparative chloroplast genomic analyses between these two species.

Nucleotide diversity (Pi) was calculated for *S. ensifolium* and *S. crotonoides* using DnaSP v6.12.03 [26]. A sliding window analysis was performed with a window size of 600 bp and a step size of 200 bp and *S. ensifolium* as the standard.

To compare the structural variation of the chloroplast genomes, the positions of boundaries between single-copy (LSC, SSC) and inverted repeat (IRA, IRB) regions were analyzed using IRscope [1]. These boundaries were designated as JLB (LSC-IRB), JSB (IRB-SSC), JSA (SSC-IRA), and JLA (IRA-LSC). The distance between each boundary and the nearest or overlapping gene was calculated.

## Supplementary Information

Supplementary Material 1.

## Data Availability

The complete chloroplast genome sequence of *Solanum ensifolium* is available for download from GenBank (NCBI) under the accession number PP744563.

## Abbreviations

BI: Bayesian Inference
F: Forward repeat
IR: Inverted Repeat
JLA: Boundary between LSC and IRA
JLB: Boundary between LSC and IRB
JSA: Boundary between SSC and IRA
JSB: Boundary between SSC and IRB
LSC: Large Single Copy
ML: Maximum Likelihood
ORF: Open Reading Frame
P: Palindrome repeat
R: Reverse repeat
rRNA: Ribosomal RNA
RSCU: Relative Synonymous Codon Usage
SSC: Small Single Copy
SSR: Simple Sequence Repeat or Microsatellite
tRNA: Transfer RNA
