# Supplementary material for: A phoenix in the greenhouse: characterization and phylogenomics of complete chloroplast genomes sheds light on the putatively extinct-in-the-wild Solanum ensifolium (Solanaceae)
Source: BMC Plant Biol. 2025 Mar 12;25:320. doi: 10.1186/s12870-025-06338-8 (PMC11900257; doi:10.1186/s12870-025-06338-8)
Supplement: Supplementary file 1 — Supplementary Material 1. [file 12870_2025_6338_MOESM1_ESM.docx]

Supplementary Material

**A phoenix in the greenhouse: Characterization and phylogenomics of complete chloroplast genomes sheds light on the putatively extinct-in-the-wild *Solanum ensifolium* (Solanaceae)**

Matthew R. Graham, Noorpreet Kaur, Cynthia S. Jones, Kurt Lamou and Bryan A. Connolly

*BMC Plant Biology*


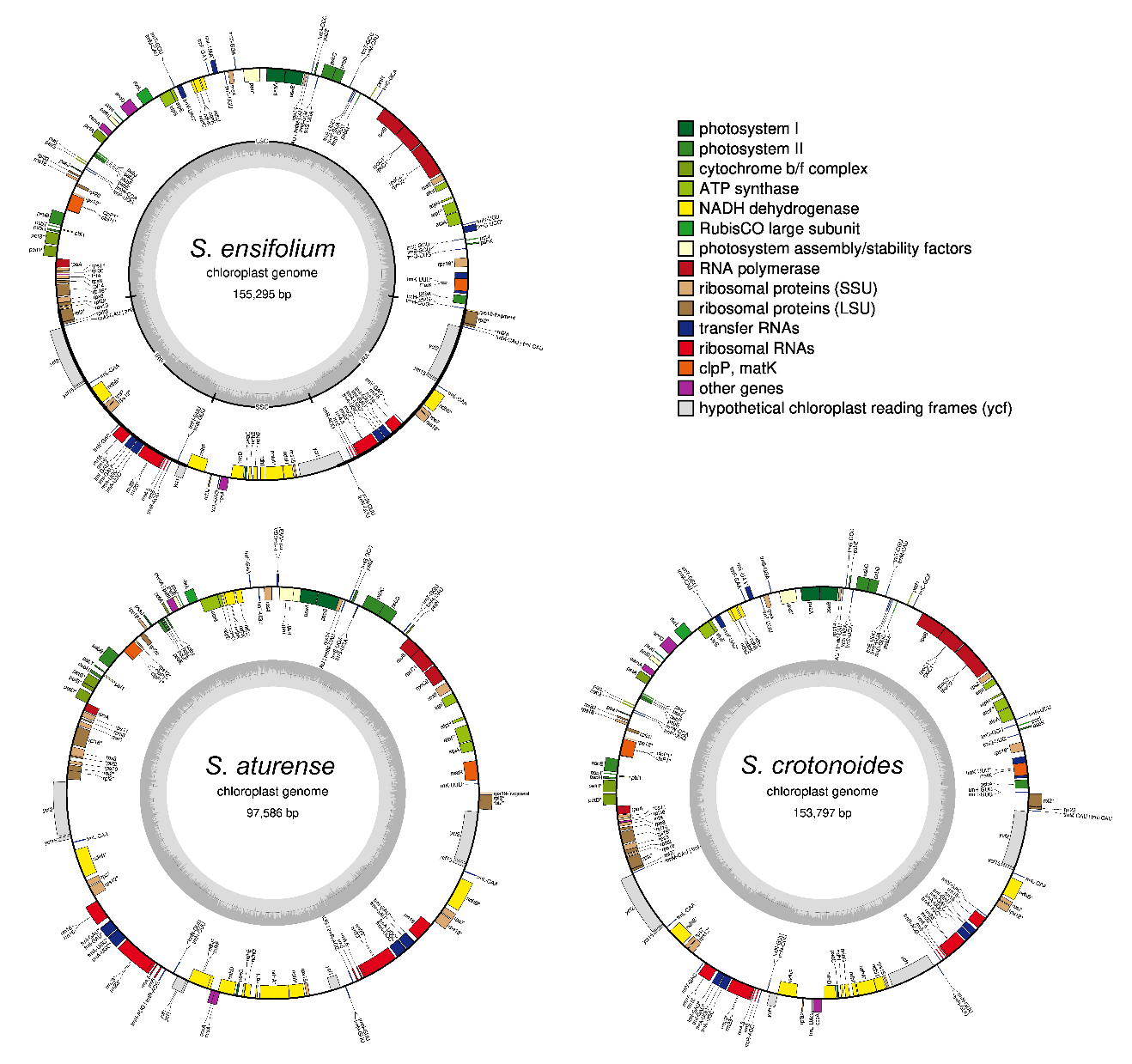


**Fig. S1.** Circular comparison of *Solanum ensifolium* with its closest relatives for which complete chloroplast genome sequences are available, *S. aturense* and *S. crotonoides*. The chloroplast genomes are depicted with the four regions (LSC, SSC, IRA, and IRB) labeled. Genes are colored according to their functional group. Genes inside the circle are transcribed clockwise, while those outside are transcribed counter-clockwise. The innermost circle represents GC content (dark gray) and AT content (light gray).

*
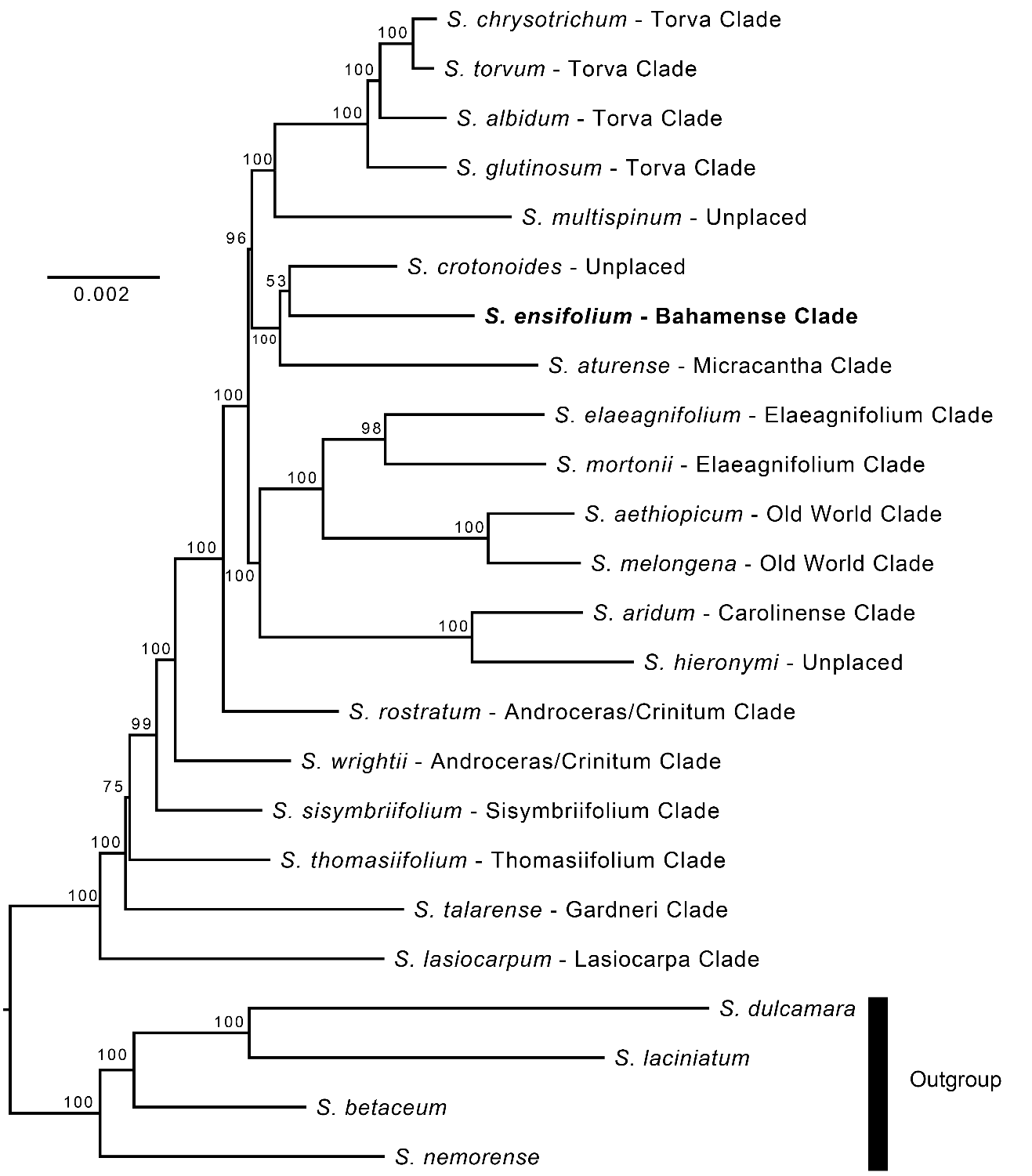
*

**Fig. S2.** Maximum likelihood (ML) phylogenetic tree inferred from complete chloroplast genome sequences exploring the placement of *Solanum ensifolium* (bold) within subgenus *Leptostemonum*. The tree includes four outgroup species for rooting purposes. Bootstrap support values are indicated at key nodes.
